# Primary hepatic lymphoma diagnosed using endoscopic ultrasound-guided liver biopsy: a case report

**DOI:** 10.1186/s13256-021-02791-9

**Published:** 2021-04-26

**Authors:** Kenichiro Nishikawa, Koji Katsuta, Syota Tanaka, Kodai Fujibe, Aiji Hattori, Yasunori Shiono, Michiaki Oiwa, Shimpei Matsusaki

**Affiliations:** 1 Department of Gastroenterology, Matsusaka Municipal Hospital, 1550, Tonomachi, Matsusaka, Mie 515-8544 Japan; 2 Department of Pathology, Matsusaka Municipal Hospital, 1550, Tonomachi, Matsusaka, Mie 515-8544 Japan; 3 Department of Gastroenterology, Suzuka General Hospital, 1275-53, Yamanohana, Yasuzukacho, Suzuka, Mie 513-8630 Japan

**Keywords:** Primary hepatic lymphoma, Endoscopic ultrasound-guided liver biopsy, Non-Hodgkin lymphoma, Case report

## Abstract

**Background:**

Because of the rarity of primary hepatic lymphomas, diagnosis of this disease entity may often be difficult, and performing a liver biopsy is the only way to establish a definitive diagnosis. Recently, endoscopic ultrasound-guided liver biopsy has emerged as a safe technique for obtaining liver tissue. However, there is no report on the use of endoscopic ultrasound-guided liver biopsy for diagnosing primary hepatic lymphomas.

**Case presentation:**

An 85-year-old Asian man was admitted to our hospital because of multiple liver lesions without any identifiable primary tumor or extrahepatic lymphadenopathy. Serum tumor markers, including alpha-fetoprotein, were in the normal range. We provisionally diagnosed the patient with a cancer of unknown primary origin with liver metastases. An endoscopic ultrasound-guided fine needle liver biopsy of the tumor in the left lobe of the liver was performed using a transgastric approach, and histology revealed a primary hepatic lymphoma of a diffuse large B-cell lymphoma type.

**Conclusions:**

Primary hepatic lymphomas are quite rare, and diagnosis is often difficult without performing a biopsy. Endoscopic ultrasound-guided liver biopsy is a useful diagnostic modality even in such cases.

## Background

A primary hepatic lymphoma (PHL) is defined as a liver-confined lymphoma without involvement of the spleen, lymph nodes, bone marrow, or other lymphoid structures [1]. PHL is a very rare malignancy and because of the rarity of this disease entity and the wide range of imaging manifestations, the diagnosis of PHL remains a challenge. Although histopathological examination is crucial, the diagnostic accuracy of traditional percutaneous liver biopsies has not always been high. Diagnosis of PHLs is often achieved only through a surgical biopsy [2–4]. Therefore, more appropriate liver biopsies may be required to obtain a diagnosis. Recently, endoscopic ultrasound-guided liver biopsy (EUS-LB) has emerged as a safe technique for obtaining liver tissue [5]. However, to date there has been no report on the use of EUS-LB in diagnosing a PHL. Here, we present a case of a PHL diagnosed using EUS-LB.

## Case presentation

An 85-year-old Asian man presented with complaints of anorexia for the few days prior to being examined. The doctor, who had previously treated the patient, confirmed hepatic and renal disorders and administered intravenous fluids, and gave a possible diagnosis of dehydration. The rest of the physical examination was otherwise unremarkable. Past medical history and family history were insignificant. He had not previously been treated with antiviral therapies, he was not jaundiced or febrile, and he had stable vital signs. His superficial lymph nodes were not palpable.

Blood tests revealed anemia with a hemoglobin level of 10.7 g/dL and a slight thrombocytopenia of 10.5 × 10^4^/µL, with a normal total and differential white blood cell count. The serum albumin level was low, at 3.4 g/dL, and the lactate dehydrogenase (LDH) level was high, at 822 IU/L. Liver function test results were abnormal, with elevated levels of alanine aminotransferase (ALT) (120 IU/L), aspartate aminotransferase (AST) (139 IU/L), alkaline phosphatase (ALP) (354 IU/L), gamma-glutamyl transpeptidase (γ-GTP) (79 IU/L), and total bilirubin (0.87 mg/dL). Renal function test results were abnormal, with elevated levels of creatinine (2.23 mg/dL) and blood urea nitrogen (32.0 mg/dL). A serological test for the hepatitis C virus (HCV) was positive, but serological tests for the hepatitis B virus (HBV) and human immunodeficiency virus (HIV) were negative.

**Fig. 1:**
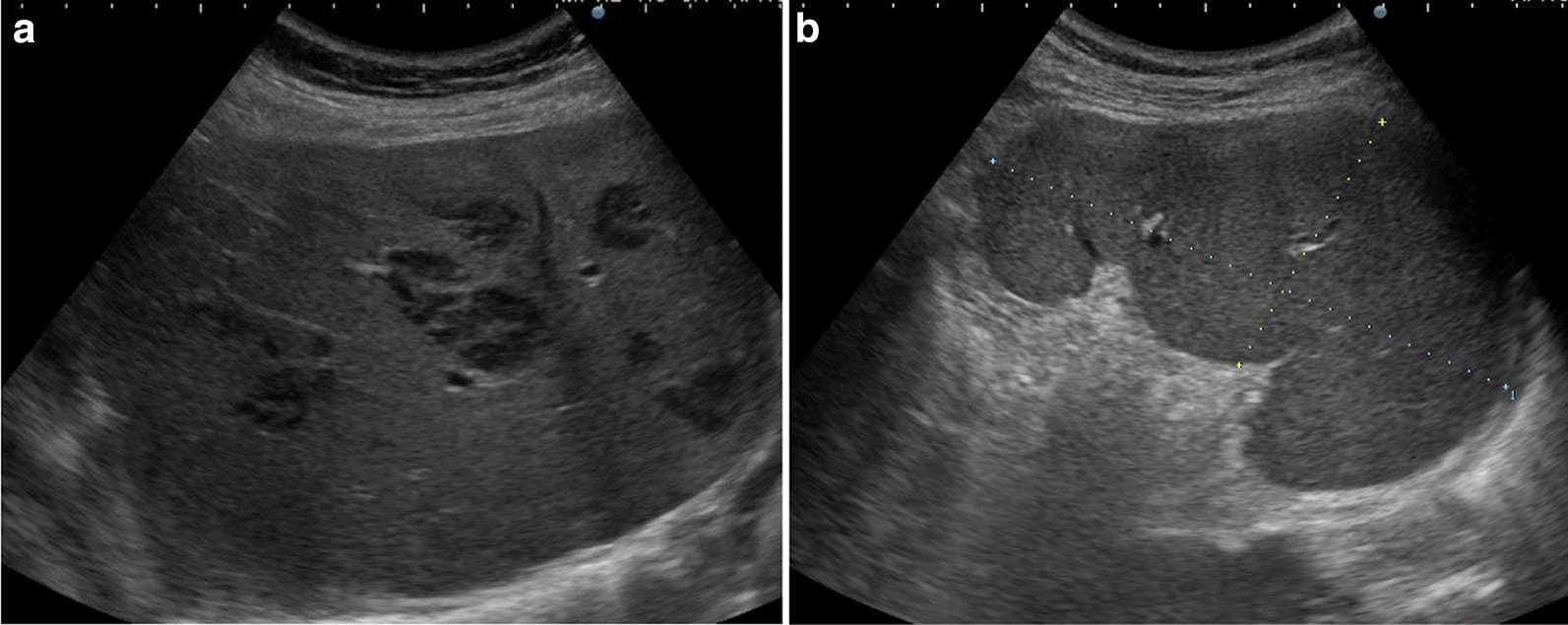
Ultrasound findings. **a** Multiple well-defined hypoechoic hepatic nodules, **b** splenomegaly without focal lesions

**Fig. 2:**
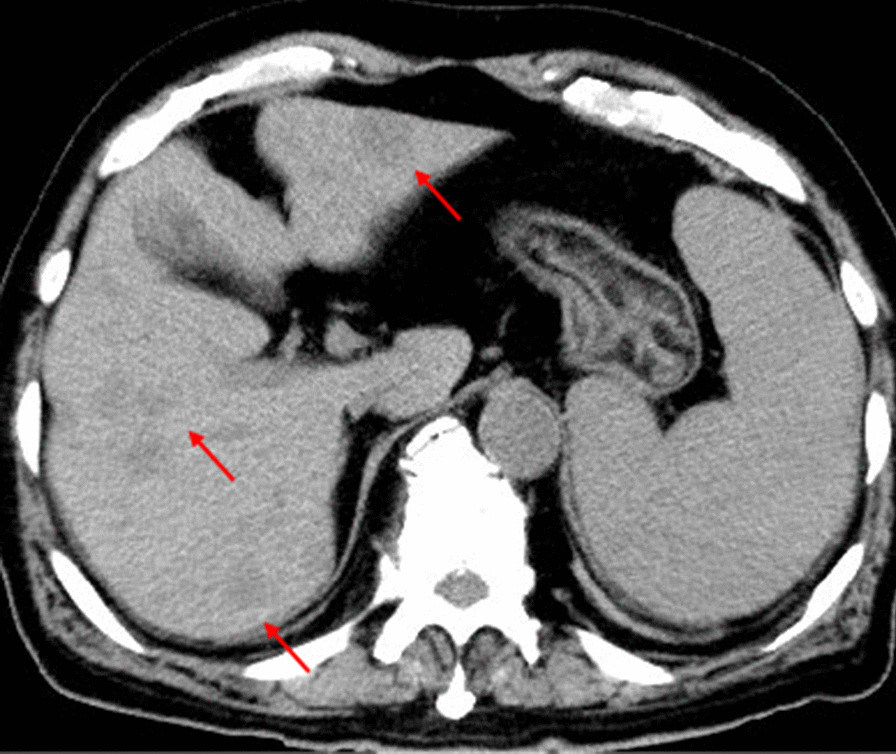
Computed tomography findings. Multiple hypoattenuating hepatic nodules without a dominant mass (red arrows). Splenomegaly was also seen

**Fig. 3:**
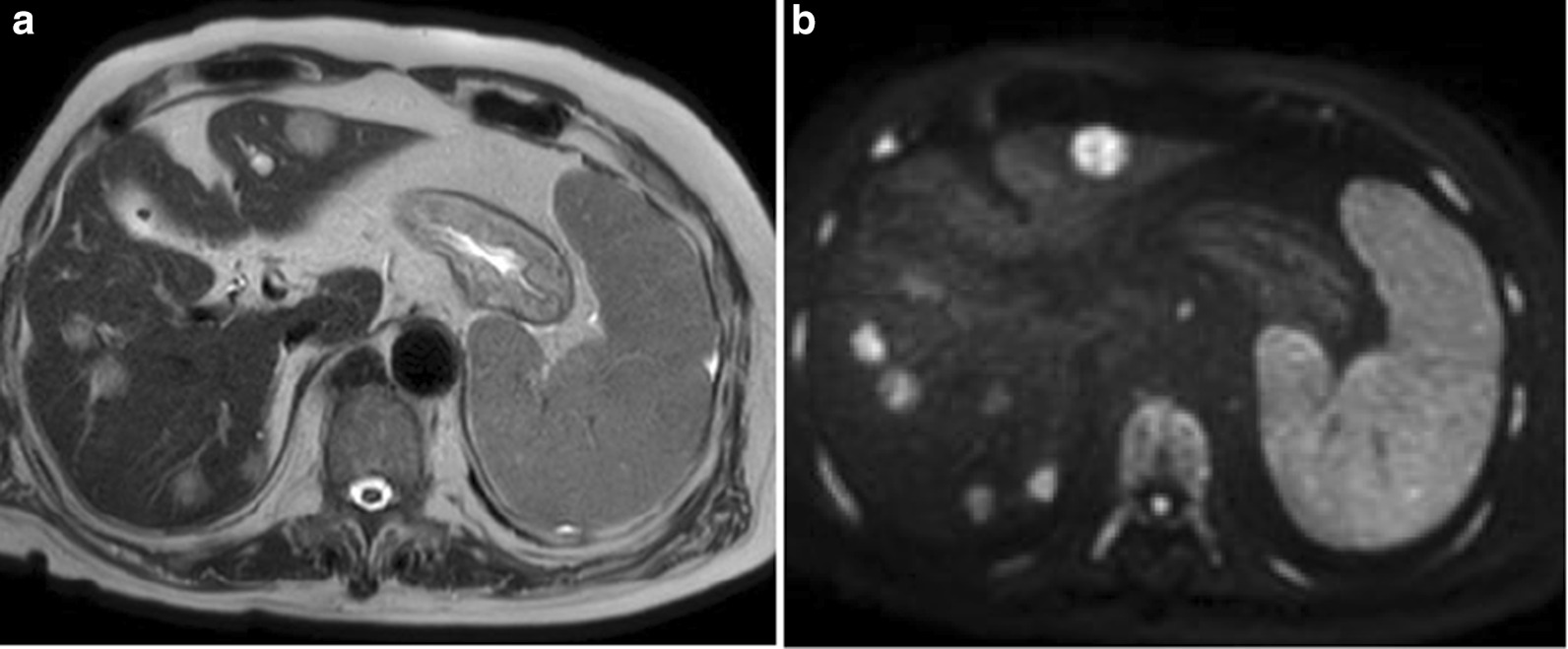
Magnetic resonance imaging findings. **a** Hepatic lesions were hyperintense on T2-weighted images, **b** marked signal restricted on diffusion-weighted images. Splenomegaly without focal lesions was also seen

An abdominal ultrasound (US) revealed multiple well-defined hypoechoic lesions in both lobes of the liver as well as splenomegaly (Fig. 1). Non-contrast-enhanced computed tomography (CT) scans of the abdomen and pelvis showed that the liver lesions were hypodense (Fig. 2). Because of renal dysfunction, the patient was unable to undergo a contrast-enhanced CT. Abdominal magnetic resonance imaging (MRI) of the hepatic lesions showed hyperintense signals on T2-weighted imaging (T2WI) and marked signal restriction on diffusion-weighted imaging (DWI) (Fig. 3). Although the patient had splenomegaly, there were no lesions within the splenic parenchyma visible upon US, CT, or MRI. The mesenteric, para-aortic, and retroperitoneal lymph nodes were not enlarged. The image was suggestive of metastatic liver tumors with liver cirrhosis without any identifiable primary tumor or extrahepatic lymphadenopathy.

Further workups were carried out to exclude a possible primary origin. Levels of tumor markers alpha-fetoprotein (AFP), carcinoembryonic antigen (CEA), carbohydrate antigen 19-9 (CA19-9), squamous cell carcinoma (SCC) antigen, pro-gastrin-releasing peptide (Pro-GRP), and prostate-specific antigen (PSA) were within the normal range. The upper gastrointestinal endoscopy and total colonoscopy reveal no abnormality. CT scans of the chest and neck were negative for primary pulmonary lesions, and the hilar, mediastinal, and cervical lymph nodes were not enlarged. Based on these findings, we provisionally diagnosed the patient as having a cancer of unknown primary origin with liver metastases.

**Fig. 4:**
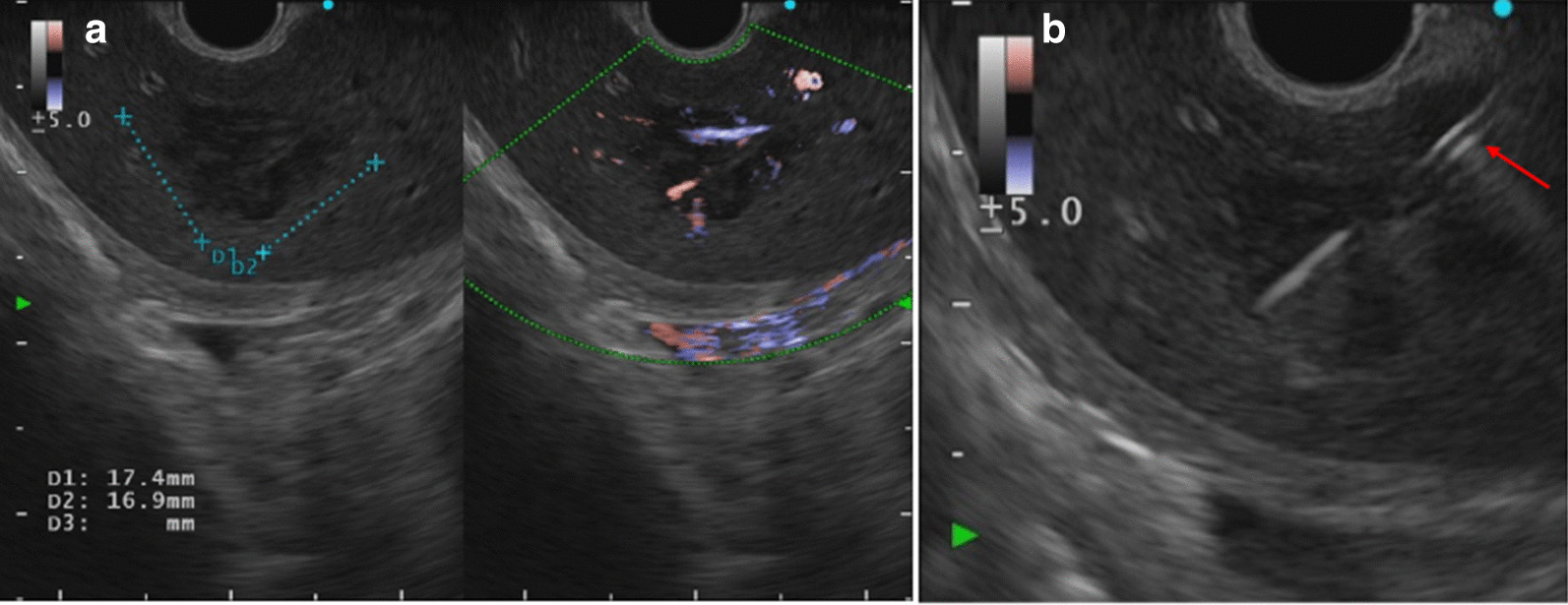
Endoscopic ultrasound findings. **a** Hypoechoic nodule of the left hepatic lobe (17.4 × 16.9 mm), **b** endoscopic ultrasound-guided liver biopsy was performed using a 19-gauge needle (red arrow)

**Fig. 5:**
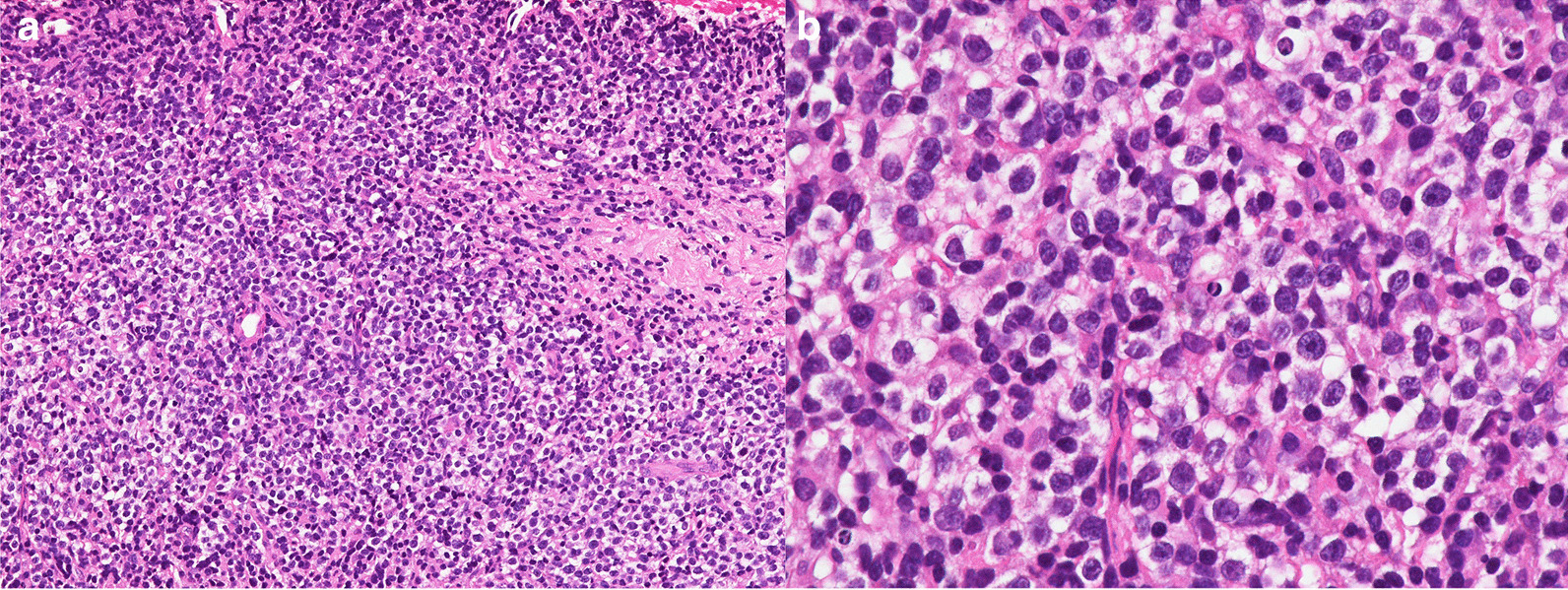
Hematoxylin and eosin stain of the tumor tissue at biopsy, revealing diffuse proliferation of large neoplastic lymphoid cells. Magnification: **a** ×100, **b** ×400

**Fig. 6:**
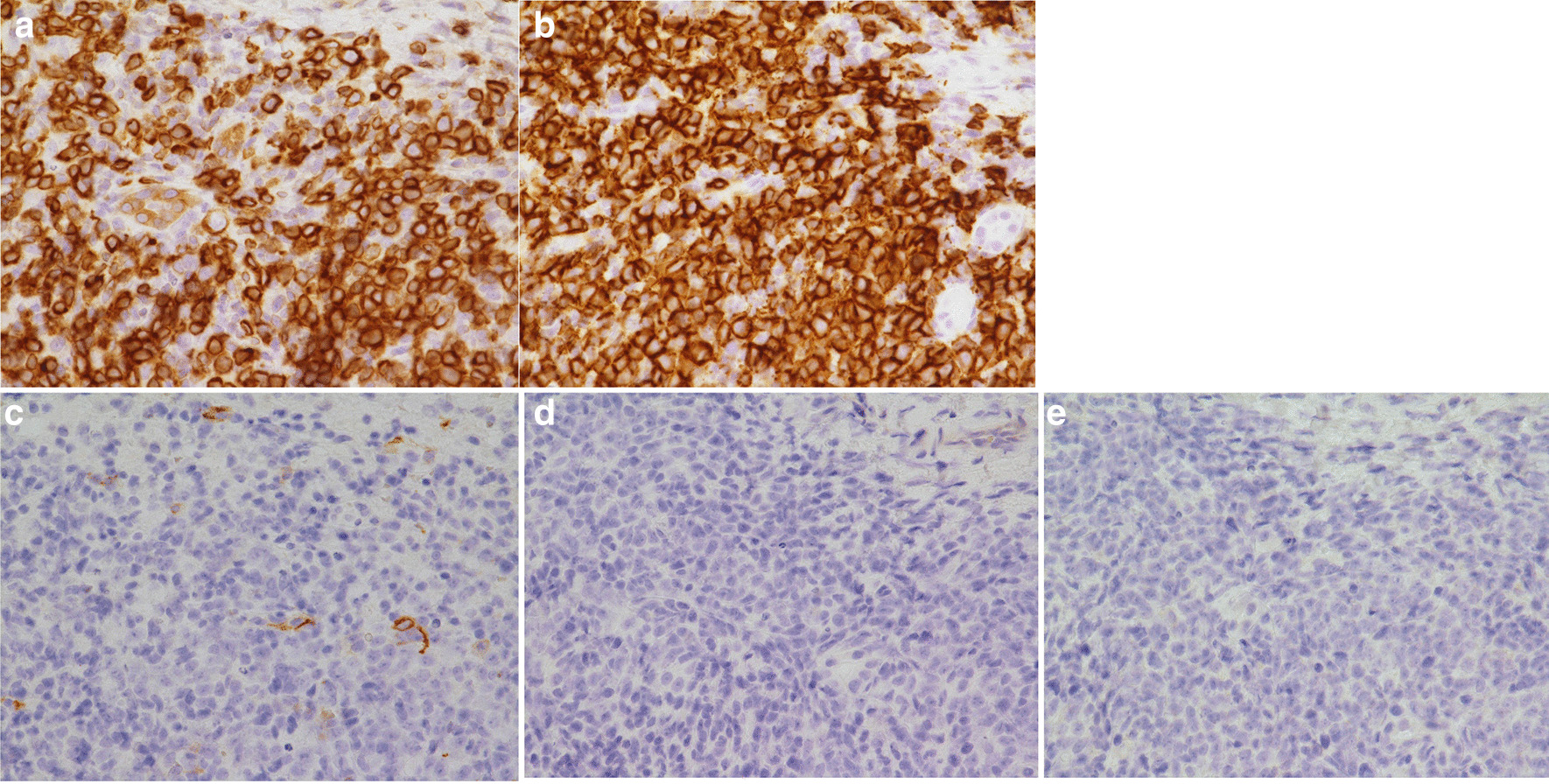
Immunohistochemical staining of the tumor tissue. **a**,** b** Positive reactivity to Bcl-2 and CD 20, **c** negative reactivity to CD56, **d** negative reactivity to synaptophysin, **e** negative reactivity to chromogranin A. Magnification **a**–**e** ×400

Endoscopic ultrasound (EUS) revealed a 17.4 × 16.9-mm almost hypoechoic nodule in the left lobe (Fig. 4a). A 19-gauge fine needle biopsy (FNB) was performed one time on the tumor in the left lobe of the liver using a transgastric approach (Fig. 4b). The needle, with suction applied using a 20-mL syringe, was moved through the entire diameter of the tumor lesion for ten strokes, and the needle was then withdrawn from the lesion. The apparatus used was a convex-type EUS gastrovideoscope (model GF-UCT260; OLYMPUS Co., Ltd., Tokyo., Japan) and a EUS processor (EU-ME2; (OLYMPUS Co., Ltd.) at a frequency of 7.5 MHz. The needle used for the EUS-LB was a disposable 19-gauge needle (Acquire; Boston Scientific Co., Natick, MA, USA). Histological analysis showed diffuse proliferation of large atypical lymphoid cells (Fig. 5). Immunohistochemistry revealed that the atypical lymphoid cells were largely positive for CD20 and Bcl-2 and negative for CD3 (Fig. 6a, b). The results of additional staining of these cells for CD56, synaptophysin, and chromogranin A were negative (Fig. 6c–e). We could therefore exclude the possibility of a small cell carcinoma.

Histopathology of the liver tissue was consistent with a diffuse large B-cell lymphoma (DLBCL). The serum soluble interleukin-2 receptor (sIL-2R) level was extremely high at 25,700 U/mL after the biopsy. Finally, we diagnosed a PHL. Although we recommended that the patient consult with a hematologist, he chose best supportive care but refused hospice care. He received regular visits from palliative care doctors and daily nursing care. He died of the disease at his home 26 days after being discharged from our hospital.

## Discussion

Our case highlights the potential utility of EUS-LB in the diagnosis of a PHL. A PHL is a very rare malignancy that constitutes about 0.016% of all non-Hodgkin lymphomas (NHLs) [6]. The usual histological type is a DLBCL [7]. In general, typical abnormalities revealed by laboratory test results associated with PHLs include anemia, neutropenia, hypercalcemia, and variably elevated serum LDH and bilirubin levels, as well as elevated ALP and aminotransferase activities. Tumor markers such as AFP and CEA are usually within the normal range [8–10]. Normal levels of serum tumor markers are useful in distinguishing PHLs from hepatocellular carcinomas and metastatic liver cancer.

The imaging features of hepatic lymphomas are non-specific, and PHLs can appear as solitary discrete lesions (60%) or as multiple lesions (35–40%). On US images, hepatic lymphoma lesions are usually well defined and appear markedly hypoechoic or anechoic. On MRI, the lesion tends to show hyperintense signals on T2WI and a signal restriction on DWI [11]. Retrospectively, our case was largely in line with laboratory and radiological findings described in the literature. The exact cause of PHLs is poorly understood. Several recent etiological studies have shown that there is a strong association between HCV infection and PHLs, especially in B-cell NHL. Page et al. [12] described 23 cases of primary hepatic DLBCLs and one case of a mucosa-associated lymphoid tissue (MALT) lymphoma, with six of the ten cases of DLBCL (60%) having HCV infections. Kikuma et al. [13] reported 20 cases of primary hepatic B-cell lymphoma, of which eight of 12 the DLBCL cases (66.7%) and two of eight MALT lymphoma cases (25%) had serum anti-HCV antibodies and HCV RNA. Our patient was HCV-positive, and this viral infection could be a probable risk factor for PHL.

A liver biopsy is the only way to establish a definitive diagnosis of a PHL. Because of the presence of a large area of necrosis, results from traditional percutaneous liver biopsies for PHLs have not always been accurate [2]. Recently, EUS-LB has emerged as an alternative technique to obtain liver tissue with a low adverse event profile and to provide adequate tissue yields for evaluation of liver disease [5]. During the procedure, tissue sampling is performed under real-time sonographic imaging, thereby avoiding the risk of puncturing large vessels or other organs. Other potential benefits include patient comfort, with sedation provided during the procedure. The most common adverse event of percutaneous liver biopsies is pain, which can be located at the biopsy sight or radiate to the right shoulder [14]. We offered several biopsy options, including a US- or CT-guided percutaneous liver biopsy, and the patient always preferred the least painless procedure. Moreover, it is known that the left hepatic lobe can be relatively easily visualized using EUS because the left hepatic lobe lies close to the stomach. In our case, one of these liver tumors was found in the left hepatic lobe on pre-procedural evaluation. Thus, we took into consideration all of the various factors, including the patient’s condition, and decided to perform the less invasive EUS-LB. Ching-Companioni et al. [15] determined that use of the 19-gauge FNB needle was an improvement over the traditional 19-gauge fine needle aspiration (FNA) needle used in EUS-LB. In this case, biopsy samples obtained with the 19-gauge FNB needle were adequate for histological assessment, and there were no technical failures or procedure-related adverse events. By a combination of rapid on-site cytologic evaluation, we were able to determine the quantity and quality of the tissue samples in real time, resulting in fewer required punctures and reduced total procedure time, which was 22 min.

Poor prognostic features can include advanced age, constitutional symptoms, bulky disease, unfavorable histologic subtypes, elevated levels of LDH and a high proliferation rate, cirrhosis, and comorbid conditions [16–18]. Unfortunately, our patient fulfilled some of these features and had an extremely poor outcome. Our case is the first report of a PHL being diagnosed using EUS-LB.

## Conclusion

Primary hepatic lymphomas are quite rare, and diagnosis is often difficult without a biopsy. EUS-LB is a useful diagnostic modality, even in such a rare case.

## Data Availability

Not applicable.

## Abbreviations

AFP: Alpha-fetoprotein
ALP: Alkaline phosphatase
ALT: Alanine aminotransferase
AST: Aspartate aminotransferase
CA19-9: Carbohydrate antigen 19-9
CEA: Carcinoembryonic antigen
CT: Computed tomography
DLBCL: Diffuse large B-cell lymphoma
DWI: Diffusion-weighted imaging
EUS: Endoscopic ultrasound
EUS-LB: Endoscopic ultrasound-guided liver biopsy
FNA: Fine needle aspiration
FNB: Fine needle biopsy
γ-GTP: Gamma-glutamyl transpeptidase
HBV: Hepatitis B virus
HCV: Hepatitis C virus
HIV: Human immunodeficiency virus
LDH: Lactate dehydrogenase
MALT: Mucosa-associated lymphoid tissue
MRI: Magnetic resonance imaging
NHL: Non-Hodgkin lymphoma
PHL: Primary hepatic lymphoma
Pro-GRP: Pro-gastrin-releasing peptide
PSA: Prostate-specific antigen
SCC: Squamous cell carcinoma
T2WI: T2-weighted imaging
sIL-2R: Soluble interleukin-2 receptor
US: Ultrasound
